# Systematic comparison of SCOP and CATH: a new gold standard for protein structure analysis

**DOI:** 10.1186/1472-6807-9-23

**Published:** 2009-04-17

**Authors:** Gergely Csaba, Fabian Birzele, Ralf Zimmer

**Affiliations:** 1 Practical Informatics and Bioinformatics, Department of Informatics, Ludwig-Maximilians-Universität München, Amalienstrasse 17, D-80333 Munich, Germany

## Abstract

**Background:**

SCOP and CATH are widely used as gold standards to benchmark novel protein structure comparison methods as well as to train machine learning approaches for protein structure classification and prediction. The two hierarchies result from different protocols which may result in differing classifications of the same protein. Ignoring such differences leads to problems when being used to train or benchmark automatic structure classification methods. Here, we propose a method to compare SCOP and CATH in detail and discuss possible applications of this analysis.

**Results:**

We create a new mapping between SCOP and CATH and define a consistent benchmark set which is shown to largely reduce errors made by structure comparison methods such as TM-Align and has useful further applications, e.g. for machine learning methods being trained for protein structure classification. Additionally, we extract additional connections in the topology of the protein fold space from the orthogonal features contained in SCOP and CATH.

**Conclusion:**

Via an all-to-all comparison, we find that there are large and unexpected differences between SCOP and CATH w.r.t. their domain definitions as well as their hierarchic partitioning of the fold space on every level of the two classifications. A consistent mapping of SCOP and CATH can be exploited for automated structure comparison and classification.

**Availability:**

Benchmark sets and an interactive SCOP-CATH browser are available at .

## Background

The classification and comparison of the more than 50'000 protein structures deposited in the PDB [1] (January 2009) is an essential step to extract valuable knowledge from protein structure data. Today, the two most prominent protein structure classification schemes are SCOP [2] and CATH [3]. Both partition proteins into domains. These domains are classified in a hierarchical manner: SCOP sorts protein domains into classes, folds, superfamilies and families while the four major levels of CATH are class, architecture, topology and homologous superfamily. The SCOP database is mainly based on expert knowledge and, on the first level of the hierarchy, defines four major classes namely all *α*, all *β*, *α*/*β *as well as *α *+ *β *describing the content of secondary structure elements in the domain. According to the SCOP authors, domains in a common fold have the same major secondary structures in the same arrangement with the same topological connections. In the same superfamily, domains share low sequence identities but their structures and, in many cases, functional features suggest that a common evolutionary origin is probable while domains clustered in the same family are likely to have a common evolutionary origin based on sequence similarity or functional evidence.

The building process of CATH contains more automatic steps and less human intervention compared to SCOP. Analogous to SCOP, CATH starts at the class level defining three major classes of secondary structure content (all *α*, all *β *and *α*/*β*). The second layer, called architecture, clusters domains with common general features with respect to the overall protein-fold shape but does not take connectivity into account. The topology level is analogous to the SCOP fold level and groups structures that have a similar number and arrangement of secondary structure elements with the same connectivity. The last (major) level, homologous superfamily, clusters domains with a high structural similarity and similar functions, which suggest that they may have evolved from a common ancestor.

In the last years, SCOP and CATH have been used to address various questions in structural biology and are further employed as training and gold-standard databases making them invaluable resources in structural bioinformatics. They have been used to study the interplay of protein structure and protein sequence evolution [4,5] or to explore the connection between alternative splicing and protein structure evolution [6].

Besides those analyses, they are often used in the context of automatic protein structure classification and protein structure prediction when training and evaluating the respective methods. Automatic protein structure classification (given the resolved structure) has become an important topic with the faster growing number of PDB structures in order to analyze structural and functional features of proteins. Methods which are specifically suited for an accurate and automatic assignment of structures to their respective class often use SCOP or CATH as reference and template datasets or to evaluate their performance. Among those methods which heavily use SCOP or CATH for structure prediction from the sequence are AutoSCOP [7] and PFRES [8]. Examples for methods to compute protein structure alignments and to predict similarities between structures are Vorolign [9], PPM [10], FatCat [11] or TM-Align [12]. In the context of machine learning, various methods to discriminate between structural classes defined by SCOP or CATH, e.g. using Support Vector Machines [13,14], have been published.

Also, various methods which aim at the prediction of a proteins structure from the sequence [15] have been developed in the last years, especially in the context of the CASP experiments reviewed e.g. in [16] (and references within) where reference databases such as SCOP and CATH are used in the prediction and the assessment phase. Of course, differences in the reference sets will inevitably lead to differences in the assessment reflecting the performance with respect to the criteria used to construct the reference sets.

Although the two hierarchies have become the gold standard in the field, their goals and the methods used to classify structures are not the same which leads to different classifications of the same protein. Differences are found with respect to the domain partitioning of the protein chain, as well as in the classification of a domain into its corresponding structure class. Differences and similarities between SCOP and CATH have already been evaluated [17,18] and those analyses allowed for valuable insights into the problems and challenges of classifying protein structures. Since the most recent study [18] the number of protein structures available in the PDB has more than doubled. This fact may have also increased the problem classifying all known structures in a consistent manner. In contrast to previous studies, we will focus on the extraction of consensus classifications based on the detailed comparison of the two hierarchies which should be a useful resource for gaining insights in functional and evolutionary relationsships and for (machine learning) methods for protein structure classification and prediction. In more detail, we propose a new approach to compare SCOP and CATH on the different levels of the two hierarchies using a similarity measure for sets of domains. Based on an initial mapping of individual domains defined in both hierarchies and on the similarity of two sets of domains, we identify for each set from one hierarchy the corresponding overlapping set(s) from the other hierarchy. This allows to map sets of domains on different levels of SCOP and CATH and to analyze the differences and similarities of the two hierarchies in detail.

SCOP and CATH are often used as 'standard of truth' datasets and inconsistencies and differences in the hierarchies unavoidably lead to problems in the training phase (since wrong or misleading concepts are learned) as well as in the testing/benchmarking phase. Proteins classified to be different by one hierarchy may indeed be similar according to the other classification leading to an overestimation of the errors made. To overcome those problems, we extract sets of pairs of protein domains from our SCOP-CATH mapping which are consistently classified in both hierarchies. Those pairs represent a novel and comprehensive benchmark (training) set which allows for a more consistent evaluation (and training) of protein structure comparison and protein structure prediction methods.

Finally, we utilize our mapping as orthogonal evidence in order to identify potential connections between different folds in one hierarchy which may be revealed via a connection of the two folds suggested by the respective other hierarchy. Such connections between different folds (which are supposed not to be evolutionary related due to the SCOP or CATH definition) provide interesting starting points to further analyze the protein sequence-structure space.

## Results and Discussion

### Datasets

For our analysis we use the most current version of SCOP (1.73, September 2007) as well as CATH version 3.1.0 (January 2007) which contains a similar number of proteins. The mapping containing the more recent CATH version 3.2.0 can be found on the supplementary website at . The website and the benchmark datasets will be updated regularly when new versions of SCOP and CATH are released. SCOP 1.73 contains 34'495 proteins deposited in the PDB (97'178 domains) which are classified into 11 classes, 1'283 folds, 2'034 superfamilies and 3'751 families. CATH comprises 30'028 PDB proteins which are partitioned into 93885 domains and sorted into 4 classes, 40 architectures, 1'084 topologies and 2'091 homologous superfamilies. The union set of the proteins in the two classification schemes contains 36'970 proteins. 27'553 PDB proteins are classified in both hierarchies. Please note that throughout this article we regard the following levels of SCOP and CATH to correspond to each other: SCOP family/superfamily ↔ CATH homologous superfamily, SCOP fold ↔ CATH topology, SCOP class ↔ CATH class.

### Detailed Comparison of SCOP and CATH

In the following we present the results of our analysis of similarities and differences between SCOP and CATH. We will first discuss the results of mapping the different domain definitions of SCOP and CATH onto each other, showing that there are surprisingly large differences between SCOP and CATH with respect to their domain definitions. We will then use the set of mappable domains (for which domain definitions largely agree), restrict the respective hierarchies to those domains and compute the mapping of inner nodes of the two restricted hierarchies. We then analyze this mapping of inner nodes in detail which turns out to be very complex indicating many inconsistencies between SCOP and CATH. For interesting examples see Additional File 1. The usefulness of the SCOP-CATH mapping is demonstrated by two applications.

Our analysis depends on whether we map SCOP to CATH or vice versa. We present the results of the (non symmetric) mapping of SCOP → CATH in the following. The results for the mapping of CATH → SCOP are available in the supplementary material on .

#### Domain mapping

In order to analyze the different domain definitions in SCOP and CATH, we keep a domain defined in one hierarchy fixed and count how often one or more domains from the respective other classification are mapped onto it. A domain is mapped *iff *the overlap *o*, as defined in the Methods section, is greater than 0, i.e. we map all domains which have at least one residue in common with the query domain. The results are shown in Table 1 and confirm results from previous studies [17] that SCOP tends to define larger domains which may be represented by several, smaller domains in CATH.

**Table 1:** Mapping of the domain definitions of the two hierarchies

	1	2	3	4	5	6
SCOP	49'251	17'162	1'885	435	130	29
CATH	68'270	11'018	492	3	0	0

Mapping of the domain definitions of the two hierarchies. An overlap threshold > 0 is used, i.e. all domains which share at least one residue are mapped onto each other. The SCOP row shows the number of CATH domains mapped onto a single SCOP domain, while the CATH row describes the number of SCOP domains mapped onto one domain defined in CATH. A single domain in SCOP may be partitioned into up to 6 domains in CATH. Overall, about 20'000 (19'641) out of about 70'000 (68'892) SCOP domains map more than one CATH domain while about 11'500 (11'513) out of about 80'000 (79'783) CATH single domains map to more than one SCOP domain.

For our final mapping of domains we use a much more restrictive overlap threshold of *T*_*o *_= 0.8. This implies a unique and bijective mapping of domains onto each other but leaves many domains unmapped. Including protein domains which overlap to only a small extent would lead to additional problems when comparing the two hierarchies, especially since domains are also classified according to their secondary structure elements and content (see [19] for further discussion). Therefore, including secondary structure elements in the domain of one hierarchy while not including them in the other one is likely to lead to differing classifications. The strict threshold of 0.8 assures that only domains which are defined as the same parts of the protein structures in SCOP and in CATH are contained in the final dataset.

As shown in the following, differing domain assignments have a large impact on the resulting classification. Out of the 27'553 proteins which are classified in both hierarchies, for only 19'266 (about 70%) the domain definitions are similar enough leading to 56'104 domains in the final dataset (increasing up to 66'128 mappable domains with an overlap threshold *T*_*o *_> 0.5). In SCOP, those domains are classified into 11 classes, 754 folds, 1'258 superfamilies and 2'228 families which means that on the other hand, for 538 folds, 776 superfamilies and 1'523 families already the domain definitions of SCOP and CATH differ to such a large extent that they can not be meaningfully mapped onto each other. According to CATH, the proteins belong to 4 classes, 38 architectures, 736 topologies and 1'462 homologous superfamilies. Two architectures, 348 topologies and 629 superfamilies of CATH remain unmapped. Those values show a surprisingly large number of domains in either of the two hierarchies which are defined in a very different manner in the respective other classification scheme according to their domain boundaries and result in the fact that for only 70% of the proteins the classifications can be compared. Moreover also only 70% of all SCOP families and CATH superfamilies are retained in the mapping due to differing domain assignments.

#### Mapping of Inner Nodes

Given the set of mappable domains as discussed above, we computed the mapping of inner nodes of the two hierarchies as described in the Methods section. The results are shown in Table 2. Using the F-measure (see Methods) we are able to identify for every inner node of SCOP the corresponding, i.e. best fitting, node in the CATH. In such a mapping one would e.g. expect that SCOP superfamilies (and families) map best to the CATH homologous superfamily level.

**Table 2:** Mapping distribution of SCOP onto CATH nodes

F > 0	Unmapped	C	A	T	H
fold class	0	**4**	**2**	1	4
Fold	0	0	5	**504**	236
superfamily	0	0	2	32	**1'224**
Family	0	0	1	9	**2'218**

F > 0.8	Unmapped	C	A	T	H

fold class	8	**2**	**0**	0	1
Fold	125	0	4	**439**	177
superfamily	236	0	1	24	**997**
Family	1'055	0	1	6	**1'166**

Number of inner nodes from a hierarchy level in SCOP mapping best to a node from some level in CATH. Consistent mapping are displayed in bold. Two different F-measure thresholds of 0 and 0.8 are shown. For example, 504 SCOP folds map best to a CATH topology node given a threshold of 0 dropping down to 439 nodes for a F-measure threshold of 0.8.

Surprisingly, when using a F-measure threshold of 0 (we map every query SCOP node onto the CATH node with maximal F-measure), the mapping of inner nodes and, therefore, the partitioning of the fold space according to SCOP and CATH appears to be more complicated than expected and many inconsistencies can actually be observed. When we require a certain quality for a mapping, i.e. setting the F-measure threshold to 0.8, a large number of inner nodes do not find a partner in the other hierarchy. SCOP and CATH therefore define their sets of domains on every level of the hierarchies and for many cases very differently and a large number of unexpected mappings (all the cases except for the cells marked bold in Table 2) can be observed. For example 240 (178) homologous superfamilies in CATH can not be mapped to a corresponding SCOP superfamily or family for a F-measure threshold of 0 (0.8). The complete mapping and the observed differences between SCOP and CATH can be interactively and comprehensively explored on .

#### Comparison of domain pairs

In order to analyze the surprisingly large number of inconsistencies between SCOP and CATH in more detail, we tested all pairs of domains in the set of mappable domains for their consistency in the respective other hierarchy. For example, we test if a pair from the same SCOP superfamily is also classified to be in the same homologous superfamily level in CATH. The results of this pairwise comparison of the two hierarchies are shown in Tables 3 and 4. This analysis reveals a very large number of domain pairs which are not classified consistently in the two hierarchies. Even on the family level, where the evolutionary relationship of the proteins should be clear, 98% of the pairs are consistently defined, more than 130'000 pairs classified into 70 different folds and 102 superfamilies are not classified in a consistent manner. More than 700'000 pairwise errors are observed on the superfamily and more than two million errors on the fold level. Table 4 allows for a more detailed analysis of the mapping between the different levels of SCOP and CATH and the errors that occur. For example 0.866% (corresponding to 70'188 pairs) of the domain pairs from the same SCOP family are classified to be in different topologies (of the same CATH class) in CATH.

**Table 3:** Inconsistencies between SCOP and CATH

	consistent	inconsistent	folds	superfamilies
family	**7'970'415**	133'335	70	102
superfamily	**8'208'965**	713'181	121	159
fold	**10'879'564**	2'389'191	84	500
class	**268'747'988**	62'849'692	745	1'258
other class	**962'011'672**	249'897'353	745	1'258

Shows the inconsistencies between SCOP and CATH with respect to the levels of the SCOP hierarchy. The second column displays the number of consistent pairs (pairs of proteins from folds, superfamilies and families in the cells marked bold in Table 4) and the third column the number of inconsistent pairs. Columns four and five display the number of distinct folds and superfamilies which account for the inconsistencies observed.

**Table 4:** Detailed mappings of domain pairs in percent from SCOP onto CATH

	outer	class	fold	superfamily	family
outer	**79.38%**	8.31%	0.99%	0.40%	0.03%
class	18.16%	**56.15%**	2.55%	1.88%	0.87%
arch	2.42%	**24.90%**	2.80%	1.27%	0.09%
top	0.04%	10.50%	**81.99%**	4.44%	0.66%
hom	0.002%	0.14%	11.66%	**92.01%**	**98.34%**

Displays the detailed mappings of domain pairs in percent from SCOP (columns) onto CATH (rows). Columns sum up to 100% and table cells marked in bold display consistent mappings. Please note that due to the very large number of pairs, even small percentage values correspond to many examples (see Table 3 for details).

Fortunately, many errors are contributed by a relatively small number of 'superfolds' (Rossmann folds, immunoglobulin and some others). Those fold classes also build clusters of similar folds which are further discussed in the context of interfold similarities below.

Nevertheless, a large number of inconsistencies can not be explained by these well known superfolds. All inconsistent pairs can be interactively explored on . An interesting example is the pair d1bbxd_ and d1rhpa_. The domains are classified to belong to two different classes in SCOP (b.34.13.1 and d.9.1.1, respectively) and are indeed very different on the structure level, but belong to the same homology level according to CATH (2.40.50.40). A second example is the pair d1ku7a_ and d1j9ia_ (classified as a.6.1.5 and a.4.13.2). The two domains are indeed structurally similar (though they have a different number of helices). They are classified as different folds SCOP but belong to the same homology level in CATH (1.10.10.10). More examples and structural superpositions of such pairs can be found in Additional file 1.

All inconsistencies will lead to problems when benchmarking automatic structure classification methods. Also, they may lead to learning wrong concepts in the training phase of machine learning methods for protein structure classification for two reasons: 1.) decision criteria are only learned with respect to one classification and 2.) criteria are ignored in the learning phase because of inconsistencies.

#### Extraction of a novel benchmark set

The pairwise comparison also allows us to extract sets of domain pairs which are consistently defined across the hierarchies and which may be used as novel benchmark sets to train and evaluate structure comparison methods. In particular, we extracted two sets of domain pairs:

• domains which are consistently defined as being similar in both hierarchies (in the following denoted as the SCOP-CATH set) corresponding to the consistent fold, superfamily and family pairs in Table 3.

• non-similar, negative domain pairs, i.e. domains in the same class, which are consistently classified into different folds.

Also, to avoid an overrepresentation of very similar domains in the dataset, we clustered the domains according to their sequence similarity. All domains with a pairwise sequence identity of more than 50% were clustered together. For each cluster we retained only one representative domain in the final benchmark set (SCOP-CATH50 set). The sets can be obtained at . We also provide additional data, i.e. the details of the clustering process, which allows users to define their own benchmark sets using different sequence identity cutoffs in case that other sequence identity thresholds are appropriate for the specific application.

Redfern et al. also used a consistent set between SCOP and CATH in benchmarking their CATHEDRAL method [13]. Our approach is designed to contain all pairs of proteins which are consistently defined between the two databases. This is an important feature for benchmarking structure classification methods in very detail on a large set of different fold topologies. In contrast, the Redfern dataset, designed for a different purpose, focuses on consistently defined superfamilies whose members overlap to at least 80%. Extracting protein pairs from these consistent superfamilies would lead to a large number of pairs in the benchmark set (up to 20% of the proteins in a superfamily) which would be actually classified inconsistently between SCOP and CATH.

Our dataset can directly be employed for training and benchmarking novel methods developed in the field on different levels of the hierarchies and therefore different levels of structural similarity. In the following, we show that this novel benchmark set allows for a much more consistent evaluation of structure comparison methods which is not biased by inconsistencies in the different gold standards.

### Applications of the SCOP-CATH mapping

In the following, we discuss the results of two applications of our detailed SCOP-CATH comparison.

#### Benchmarking Structure-Comparison methods

For benchmarking purposes, and as an examplary structure comparison method, we used the TM-align method which computes a structural alignment optimizing the TM-Score [20]. The TM-Score measures the similarity of two structures by an optimized rigid body superposition and a TM-score of above 0.4 has been described to indicate structural similarity [21,22]. TM-align has been chosen for this study since the TM-Score has already been used to discriminate between similar and non-similar proteins and should therefore allow for a good discrimination of similar and non-similar protein domains. Furthermore, the method is quite fast allowing for the computation of the more than 5'000'000 structural alignments in reasonable time.

For our analysis, we compare the performance of TM-align on the complete benchmark set with the performance on the novel benchmark set proposed in this paper. The only difference between the two sets are the pairs being evaluated. While all pairs which are similar according to SCOP are evaluated in the original setting, our novel benchmark set contains only those pairs which are consistently defined to be similar or different in both SCOP and CATH. Therefore, while the domains contained in the sets are the same, the number of pairs being compared is much smaller in our novel benchmark set than in the original set (16% of the positive pairs have been removed).

**Figure 1:**
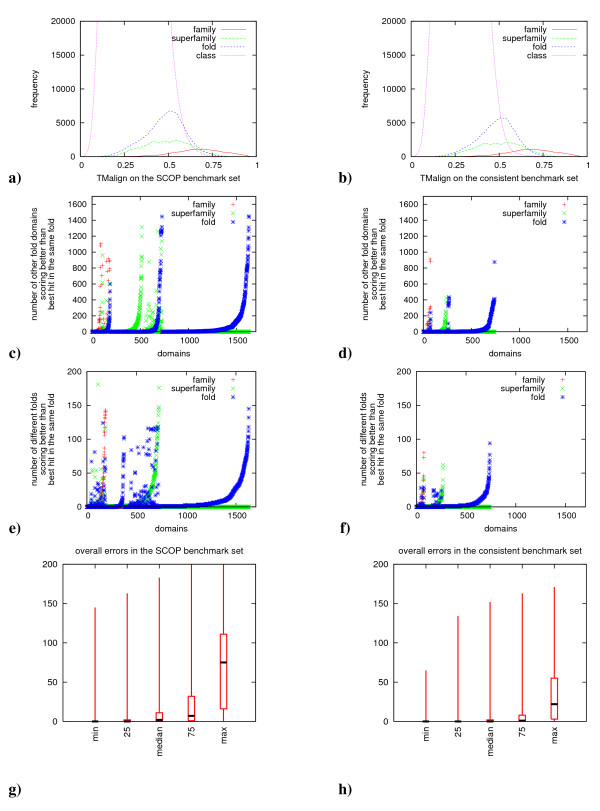
**Detailed comparison of protein structure benchmark sets**. The figure compares the performance of TM-align on the complete set of similarity relationships defined by SCOP (left column) and the performance on the novel SCOP-CATH consensus benchmark set proposed in this study (right column). For this purpose, the TM-Align performance is visualized via various plots which show in some detail the evaluation of classification errors. Panels (a) and (b) shows the distribution of scores for the various levels of the classifications. Although the fold scores are somewhat shifted to the right, the score distributions overlap significantly, which allows no clear thresholds for safe classifications of structure pairs. Panels (b)-(f) compare the various errors for the comprehensive and consensus benchmark sets. As errors we count wrong domains scored better than correct domains. The errors are significantly reduced on the consensus set (d) and (f). Finally, in panels (g)-(h) the errors (number of wrong folds scored better than certain correct folds) are summarized as boxplots. Again less errors are observed in the consensus set: whereas for the best scored correct domains quite few wrong folds are scored better in both sets, quite many better scoring but wrong folds are observed for the correct members with low scores. See main text for a more detailed description. Overall the number of errors is reduced over-proportionally (about 50% error reduction) as compared to the reduction of pairs in the consensus benchmark (about 16% pairs reduction).

In the following we will discuss the plots shown in Figure 1 which evaluate the performance of TM-align on the two benchmark sets in detail.

Plots **(a) **and **(b) **show the distribution of TM-Scores of domain pairs within the same class/fold/superfamily/family. The distributions of the scores are very similar between both sets indicating that the main properties of the benchmark sets are similar. There is no apparent bias in the benchmark set proposed here towards domains which are easier to classify and both sets appear to be equally difficult regarding their similarity relationships.

Plots **(c) **and **(d) **in row two as well as **(e) **and **(f) **in row three introduce a novel type of plot to benchmark the performance of structure comparison methods. The plots can be used for any structure comparison method to evaluate in detail the classification performance and in particular the errors made by a method. Especially, they allow to estimate the performance of a method given a template database where members of the family and superfamily are missing and analyze in detail the number of domains for which problems occur in a set of domains and also quantify the dimension of the problem. Plots **(c) **and **(d) **show the number of domains for which we observe problems according to the structural similarity detected by TM-Align. For every query domain, we show how many domains from a different fold have a higher similarity score than the highest scoring member of the domain's own family (red cross), superfamily (green x) or fold (blue star). On the x-axis we show all query domains for which we observe problems, while on the y-axis, the number of problematic cases for a query (i.e. the number of domains from different a different fold ranked higher than the own family/superfamily/fold) is plotted. For example if there are ten domains from a different fold scoring better than the most similar member from the domain's own family a red cross (at (x,10)) would be plotted. Similarly a blue dot is plotted if wrong proteins score better than a member of the query superfamily and a green x is plotted in the case of wrong domains scoring better than the own fold. Also, domains in columns which contain blue dots would not be assigned to their correct folds in the case of missing family and superfamily members since the best hit comes from a different fold.

Panels **(e)**-**(f) **in row three are similar to panels **(c)**-**(d)**, but instead of displaying the number of domains, they show the number of distinct folds (different from the domains own fold) which score better than the respective own family, superfamily or fold.

Comparing the plots that are computed based on the complete set of domain pairs (left column) with the plots computed on the benchmark set of consistent domain pairs (right column) we find that TM-Score/TM-align produces errors for only half of the domains tested and the dimension of the errors (i.e. the number of domains/folds which score better) also strongly decreases.

The only difference between the two sets tested is the removal of pairs which are inconsistently defined between SCOP and CATH. While we remove about 16% of the positive pairs (in SCOP) to obtain the consistent set, the number of errors observed is reduced by 53% (compared to 16% error reduction which would be expected when removing arbitrary pairs). Therefore, the removal of pairs which are inconsistently defined in SCOP and CATH allows to over-proportionally reduce the number of errors. Our conclusions are twofold: many errors reported for protein structure classification methods originate from pairs of domains which are similar to one another, but are classified differently by SCOP or CATH. A different set of errors results from pairs that are e.g. classified in the same family but not similar enough to be distinguished from random pairs by a structure-based comparison method. Using only pairs of domains consistently defined in SCOP and CATH allows to reduce the amount of errors significantly and to separate erroneous behaviour of a method (e.g. errors in the similarity model for protein structures implemented in a method) from problems arrising due to pairs of domains for which even gold standards and experts disagree in their classification.

This figure is completed and summarized by the plots **(g) **and **(h)**. To compute them, we sort the results obtained for every query domain according to their similarity scores. Then, we count for every member of the query fold, how many distinct other folds score better than the respective fold member (please note that every fold is counted only once even if multiple domains from a fold lead to errors). The boxplot in **(g)**-**(h) **shows the errors for five specific fold members: for the best and worst scoring fold members, as well as for the fold member placed at the 25%, median and 75% positions in the sorted list. As, unfortunately, correct fold members score quite differently, this allows to assess the overall performance of fold members by showing how often wrong members score better than the selected five fold member representatives. The boxplot now simply summarizes these numbers for all queries. Thus the boxplots give a summarized overview of the observed errors. By comparing the two boxplots for the comprehensive and the consensus sets we again find a substantial reduction of errors in the consensus set. While the number of errors for the best scoring fold member is generally small, the errors for the low-scoring fold members quickly increase in both sets, but much more drastically in the comprehensive set as compared to the consensus set. For example, if we look at the fold members scored in the lower quarter (75%) of the fold members, we find 10 different random folds before a correct domain in the original dataset and only one fold in the novel benchmark set. This again indicates, that the number of errors as well as their quantity are significantly lower in the novel benchmark set compared to the original benchmark set.

Overall, the novel benchmark set proposed here is much more consistent than the original pairwise relationships defined by SCOP. It results in a much smaller number of errors (less than half the amount of the errors in the original set). Due to the largely reduced inconsistencies the set should also be well suited for training novel machine learning algorithms for protein structure classification, since it may allow for learning more consistent concepts from the input data.

Furthermore, we expect that the new benchmark set and also the new type of plots allow for a more instructive and objective evaluation of other structure comparison methods as well. The benchmark set has already been applied to measure the performance of PPM, Vorolign and TM-align in [10].

#### Inter-Fold Similarities revealed by Consistency Checks

**Figure 2:**
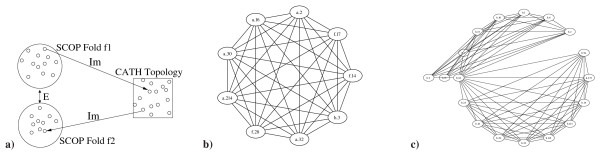
**Linking different folds via consistency checks**. **a) **Shows the method of connecting different folds in i.e. SCOP via a link proposed by the mapping of SCOP and CATH. Nodes in the graph represent SCOP folds, edges connect two nodes *iff *at least 5 members of the SCOP fold are mapped to the same CATH topology **b) **Shows the interfold similarity of *α*-hairpin proteins in SCOP which are clustered in the same fold according to CATH (1.10.287). **c) **Shows a more complicated fold graph clustering proteins of immunoglobulin (CATH 2.60.40) and jelly-roll topologies (CATH 2.60.120) in a non-clique subgraph. All fold graphs may be interactively explored on .

As a second application, we have used our mapping of the two hierarchies in order to identify similarities of different folds/topologies defined in one hierarchy which are implied by mapping them onto the same fold/topology in the respective other classification scheme. Methods to detect possible interfold similarities have already been described for example by Friedberg et al. [23] and the CATH developers [24]. Here, we do not propose a novel approach to analyze such similarities from a structural point of view but utilize the orthogonal criteria and knowledge from two curated classification schemes to identify them. More specific, we search for folds *f*_1 _defined in SCOP which map to a topology level in CATH *t *while this topology level in CATH also maps to a second fold *f*_2 _in SCOP (see also Figure 2a).

The identification of such similarities provides interesting insights into the differences and similarities of fold classifications in SCOP and CATH and further allows to identify interesting links in the fold space. In order to propose a link we currently require the existence of at least five domains, which do not share a sequence identity of more than 50%, to support the link.

This analysis reveals a large number of singletons, i.e. folds/topologies with no link to another fold. 1137 folds in SCOP as well as 904 topologies in CATH turn out to be singletons. For relatively few folds/topologies similarities with other folds are identified which are interesting cases for further analyses in the context of protein structure and sequence evolution.

For SCOP, we identified 29 subgraphs, i.e. groups of folds which are connected via a link in CATH to another fold. 18 of the groups represent graphs of size 2, i.e pairs of folds while the other 11 subgraphs connect up to 39 different folds in SCOP. The largest graph contains a cluster of SCOP folds representing domains which are classified as Rossmann fold topology (3.40.50) in CATH but are splitted into 38 different folds in SCOP. Another large cluster comprises *β*-sandwich proteins with Greek-key topology which represent a cluster of 7 folds.

Two further interesting examples are shown in Figure 2. Figure 2b shows the interfold similarity of *α*-hairpin proteins in SCOP which are clustered in the same fold according to CATH (1.10.287). Part 2c shows a more complicated fold graph clustering proteins of immunoglobulin (CATH 2.60.40) and jelly-roll topologies (CATH 2.60.120) in a subgraph which also shows that those graphs do not necessarily form a clique.

## Conclusion

Protein structure classification is an essential step towards a deeper understanding into the interplay of protein structure and protein sequence evolution. Here, we have carried out a detailed study of the similarities and differences between the two most prominent databases, namely SCOP and CATH, which have become gold standards in the field and are used in various machine learning approaches and assessments of structure prediction and classification like the CASP experiments.

We find that there are essential differences between the two classification schemes due to their way of partitioning proteins into domains (which has already been described and discussed by [25]). SCOP tends to partition domains into fewer but larger components than CATH. In total, only about 70% of the domain definitions for proteins classified in SCOP and CATH agree (at an overlap threshold of 80%) and about one third of the families in SCOP and homologous superfamilies in CATH can not be mapped on domains of the respective other hierarchy.

For the remaining set of about 20'000 proteins we then tested how well their classifications agreed with the classification in the respective other hierarchy. For this comparison we have used the F-measure to determine the similarity of two sets of domains on a specific level of two hierarchies. We find that both hierarchies show significant differences and often disagree in their way to partition the protein structure space also in cases of nearly identical domain definitions.

Given those findings and our mapping of SCOP and CATH hierarchy nodes, we extract a novel benchmark set of protein domain pairs which are defined consistently across both hierarchies. We show that benchmarking TM-align (as an examplary structure classification method) on the novel benchmark set leads to a largely improved performance in comparison to the original set where the similarities as defined with respect to SCOP. This is due to the fact that errors (proteins which are similar according to one hierarchy but separated into different classes in the other one) which occur due to inconsistencies are removed from the novel benchmark set. Therefore, the benchmark set proposed here allows for a more objective evaluation of the performance of protein structure comparison methods as the remaining errors observed are more likely to be due to the method itself. Furthermore, this set should have advantages for both, training and testing all kinds of prediction methods, especially machine learning approaches to protein structure classification since more consistent concepts may be learned in the training phase.

Finally, the mapping between SCOP and CATH provides interesting, orthogonal knowledge on the topology of the protein structure space which allows to identify non-trivial links between different folds in e.g. SCOP via their connection observed in CATH. There are some very interesting and large (up to more than 30 folds) sets which may be clustered together in SCOP according to CATH. Among them are some known clusters of folds like the Rossmann fold topology. But there are also several other clusters of folds which may be interesting starting points for a further analysis of their sequence-structure properties and may help to further understand the interplay of protein sequence and protein structure evolution, also in the context of alternative splicing [6], as different structure classifications reflect different viewpoints and criteria on structural and evolutionary similarity.

## Methods

In the following, we will handle the two hierarchies (SCOP and CATH) as labeled trees, where the leaves correspond to the domains classified in the corresponding hierarchy. Inner nodes represent sets of protein domains which are clustered together on a specific level of the hierarchy. For SCOP, inner nodes represent classes, folds, superfamiles or families. For CATH, inner nodes correspond to classes, architectures, topologies and homologous superfamilies. We denote the underlying sets of domains of the two hierarchies with *D*_1 _and *D*_2 _and the hierarchy trees themselves as *H*_1 _and *H*_2_. We further define *H*_*i *_= (*V*_*i*_, *E*_*i*_) where *D*_*i *_⊆ *V*_*i *_are the leaves of the tree. Since domain definitions in different hierarchies also may be different, we have to map the domains defined by SCOP (*D*_1_) and CATH (*D*_2_) in a first step. In a second step we will define and compute a mapping between inner nodes of the hierarchies.

### Mapping of domain assignments

A protein domain is defined as a set of segments within one protein, where a segment is defined as a consecutive part of one chain of the protein. Note that this definition also allows to define discontinuous domains and domains spanning different chains of a protein. In order to compare different domain assignments of the same protein we have to compare sets of segments. To do so, we use the sets *RP*(*d*) of residue positions for all segments of domain *d *and define the similarity of domains via the intersection of their *RP *sets. Such a mapping is not necessarily unique, i.e. it is possible that a domain in *D*_1 _maps to more than one domain in *D*_2 _or, more generally, that *n *domains in *D*_1 _correspond to *m *domains in *D*_2_. In such cases the definitions of the domains may be very different and we exclude domains from *D*_1_, *D*_2 _if their overlap *o *(see below) is smaller than a specified threshold *T*_*o*_. For two domains *d*_1 _and *d*_2 _from *D*_1 _or *D*_2_, respectively, we define the overlap *o *of two domains as:



If we use a threshold *T*_*o *_> 0.5 the mapping will be unique (but not necessarily complete).

### Mapping inner nodes of the hierarchies

While the mapping of domains is more or less trivial (except for cases where the domain definitions differ to a large extent), mapping inner nodes of the hierarchy appears to be more complicated. As already mentioned inner nodes represent sets of domains. The image of a set of domains in one hierarchy is the set of domains in the other hierarchy where *T*_*o *_exceeds a given threshold, i.e. for *S*_1 _⊆ *D*_1 _(equivalently for *S*_2 _⊆ *D*_2_) the image of *S*_1 _is defined as follows:



Further, we define the sensitivity, specificity and the F-measure of a domain mapping of two sets *S*_1 _⊆ *img*(*D*_2_) ⊆ *D*_1 _and *S*_2 _⊆ *img*(*D*_1_) ⊆ *D*_2 _on the restricted hierarchies as:



In order to map sets of domains, we search for all inner nodes *S*_1 _from hierarchy *H*_1 _and *S*_2 _from hierarchy *H*_2 _where F-measure(*S*_1_, *S*_2_) > 0, i.e. there needs to be at least one domain which occurs in both sets. The F-measure is especially useful as it accounts for a tradeoff between sensitivity and specificity. This is necessary since, obviously, the most sensitive mapping will be always the root, the most specific one the direct parent nodes of two mappable domains.

Given the F-measure for every pair of nodes which have at least one mappable domain in common, we identify the nodes in *H*_2 _which match best to a given node *n*_1 _in *H*_1_. From each path from the root to *n*_2 _in *H*_2_, only one node (the best one according to the F-measure) will be used in the mapping. Nevertheless, there may be different paths in *H*_2 _containing nodes mapped to the query node from *H*_1_. In those cases matches from different paths are also sorted according to their F-measures.

Based on those definitions we calculate for each non-leaf node *n*_1 _in *H*_1 _a sorted (by their F-measures) set *MS*(*n*_1_) consisting of all best matching non-leaf nodes for every path mapped to *n*_1 _in *H*_2_. These sets can be explored interactively via the browser at .

## Authors' contributions

GC wrote the software. GC, FB, and RZ designed research and wrote the manuscript. All authors read and approved this manuscript.

## Supplementary Material

Additional file 1 **Examples of inconsistencies between CATH and SCOP**. The file shows several interesting examples of differing classifications of protein domains in SCOP and in CATH. It contains 15 figures, each showing a structural superposition of two domains which are inconsistently classified. Click here for file
