# Supplementary material for: Systematic comparison of SCOP and CATH: a new gold standard for protein structure analysis
Source: BMC Struct Biol. 2009 Apr 17;9:23. doi: 10.1186/1472-6807-9-23 (PMC2678134; doi:10.1186/1472-6807-9-23)
Supplement: Additional file 1 — Examples of inconsistencies between CATH and SCOP. The file shows several interesting examples of differing classifications of protein domains in SCOP and in CATH. It contains 15 figures, each showing a structural superposition of two domains which are inconsistently classified. [file 1472-6807-9-23-S1.pdf]

1 Examples of inconsistencies between CATH and SCOP

2 General comments on the figures shown

In the following we show examples of structure pairs inconsistently classified in SCOP and CATH. We chose one pair of domains for each type of inconsistency that can be observed in the data. The full set of inconsistent pairs is shown and may be **interactively explored** on http://www.bio.ifi.lmu.de/SCOPCath. Structures are displayed using Jmol (http://www.jmol.org) and are superposed using TM-Align (Zhang et al. Proteins, 2004, 57, 702-710).

3 Examples for differing classifications between SCOP and CATH

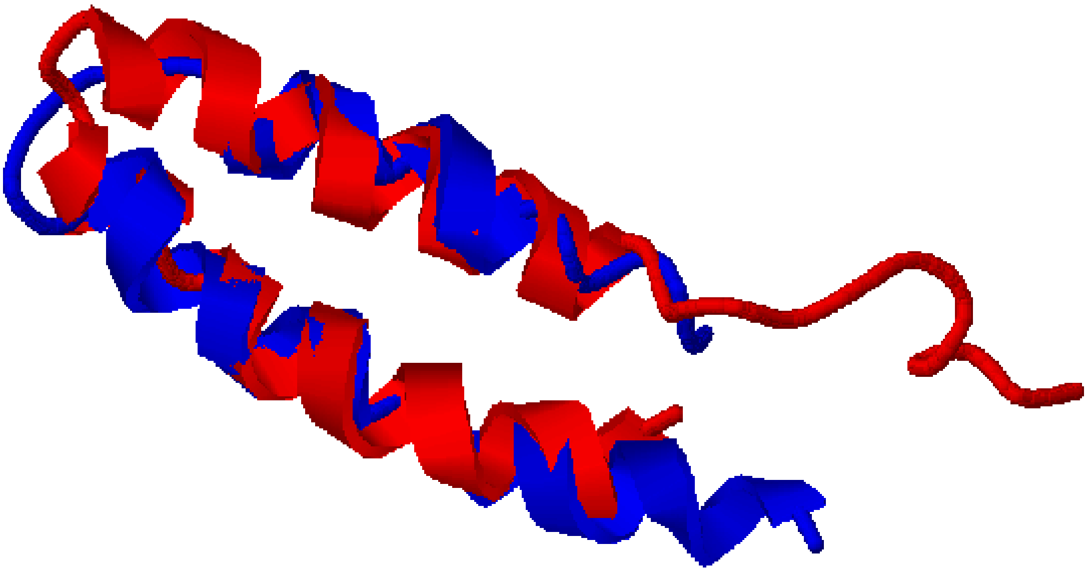

Figure 1: **Same SCOP fold, different CATH classes**: d1bsha1 (a.2.10.1) / 1bshA02 (1.20.5.440) vs. d1e52b_ (a.2.9.1) / 1e52B00 (4.10.860.10)

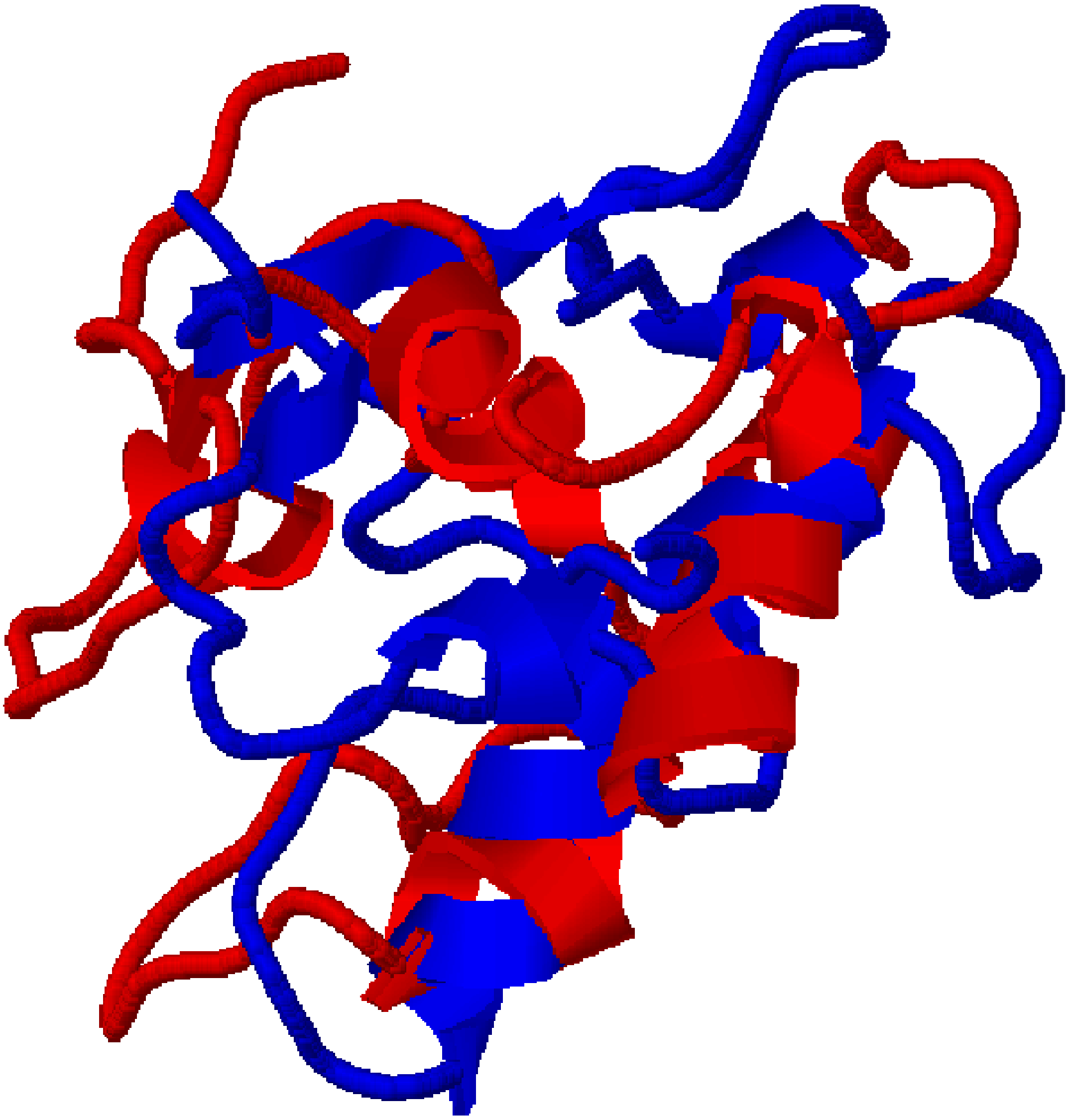

Figure 2: **Same SCOP superfamilies, different CATH classes**: d1gyob_ (a.138.1.1) / 1gyoB00 (3.90.10.10) vs. d1p2ea1 (a.138.1.3) / 1p2eA02 (1.10.1130.10)

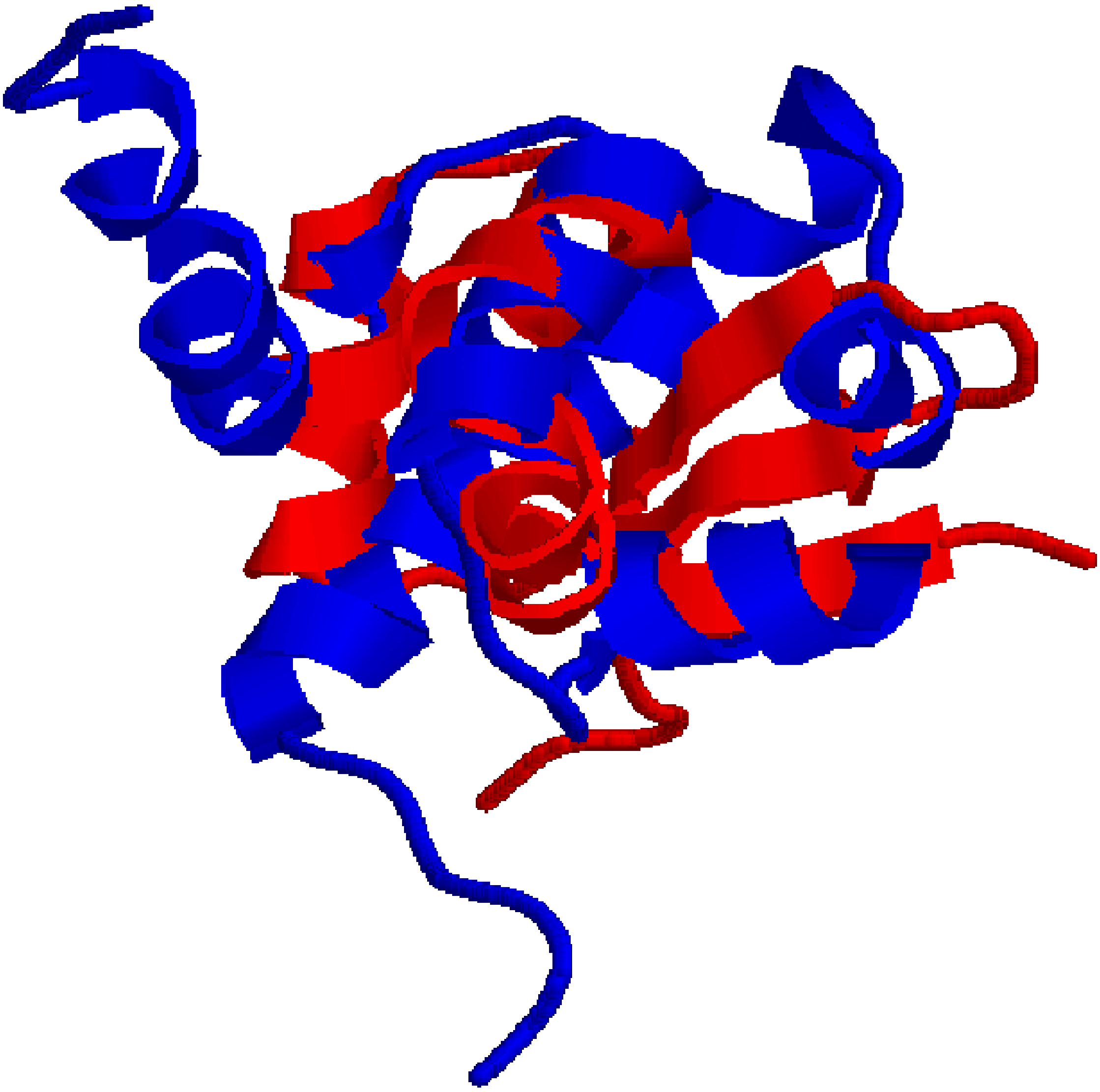

Figure 3: **Same SCOP family, different CATH classes**: d5croa_ (a.35.1.2) / 5croA00 (3.30.240.10) vs. d1rioa_ (a.35.1.2) / 1rioA00 (1.10.260.40)

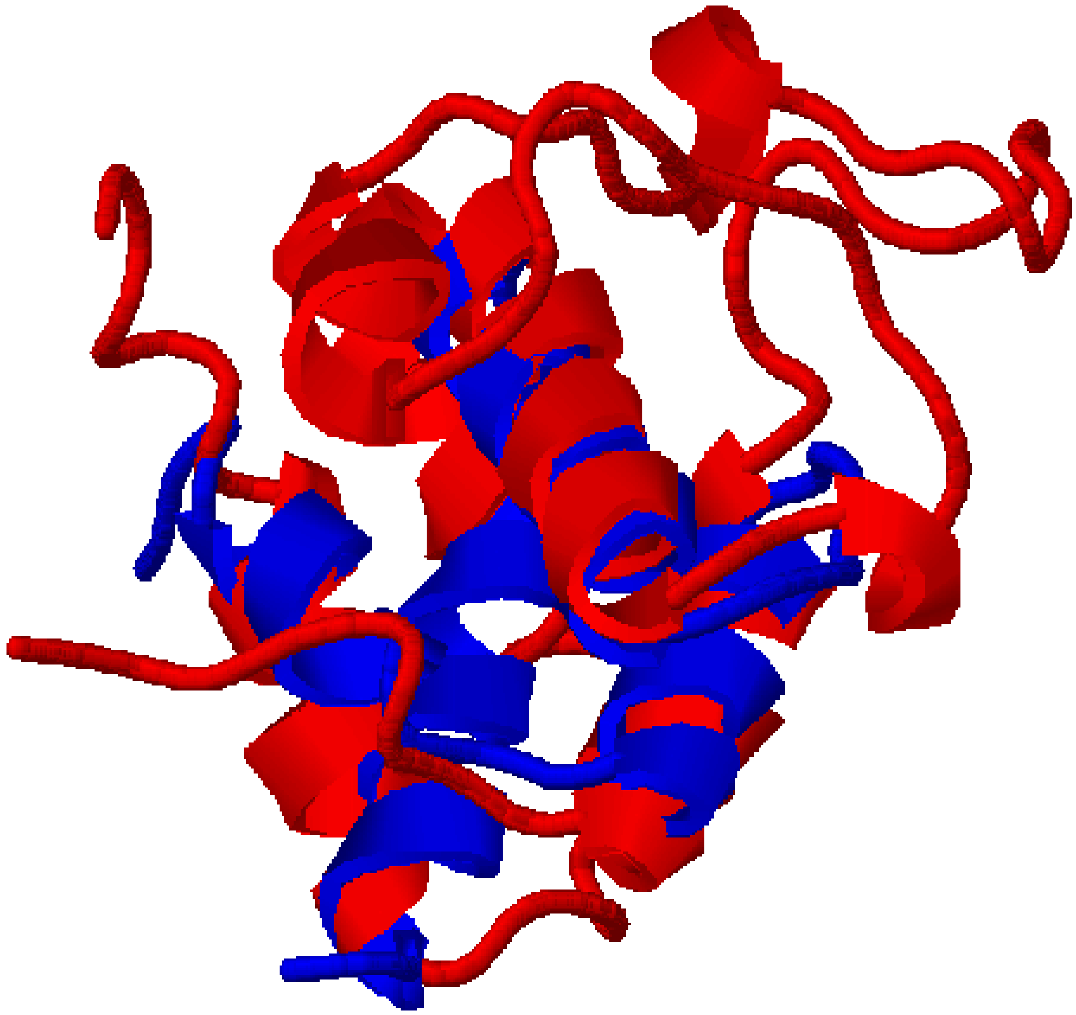

Figure 4: **Same SCOP fold, different CATH architectures**: d1v66a_ (a.140.2.1) / 1v66A00 (1.20.120.410) vs. d1wija_ (a.140.5.1) / 1wijA00 (1.10.3180.10)

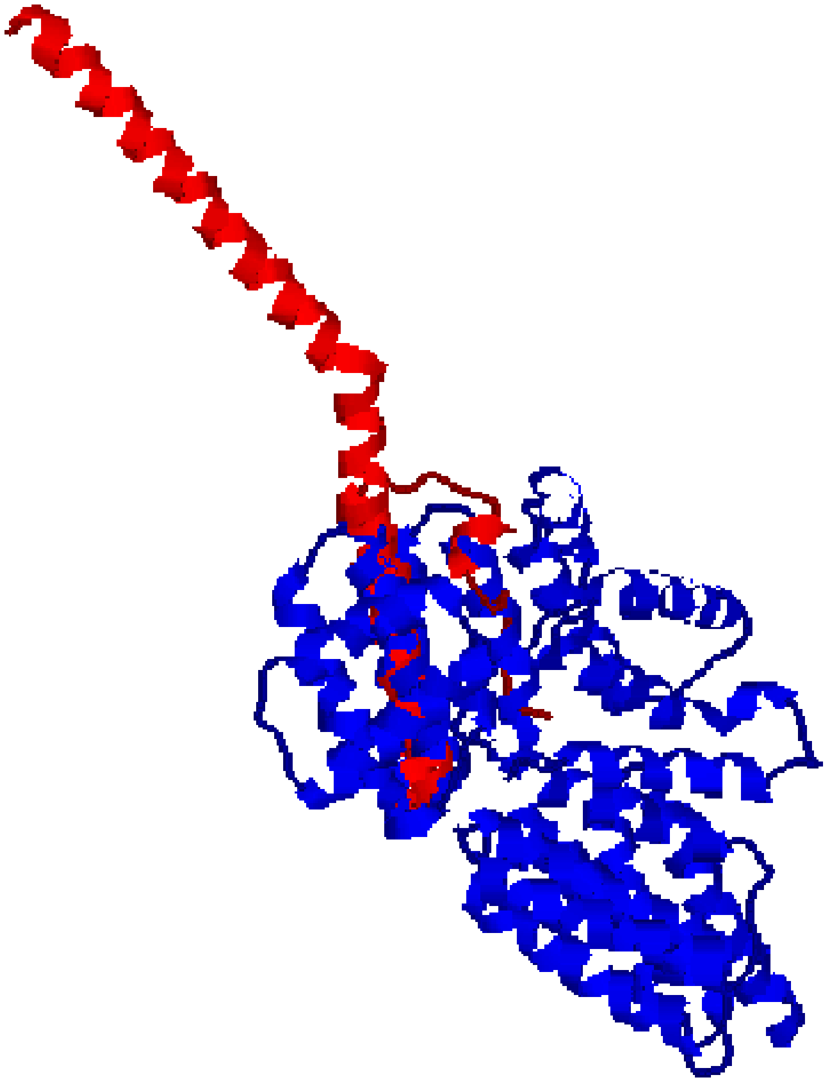

Figure 5: **Same SCOP superfamily, different CATH architectures**: d1iipa1 (a.118.8.1) / 1iipA02 (1.10.150.160) vs. d1hz4a_ (a.118.8.2) / 1hz4A00 (1.25.40.10)

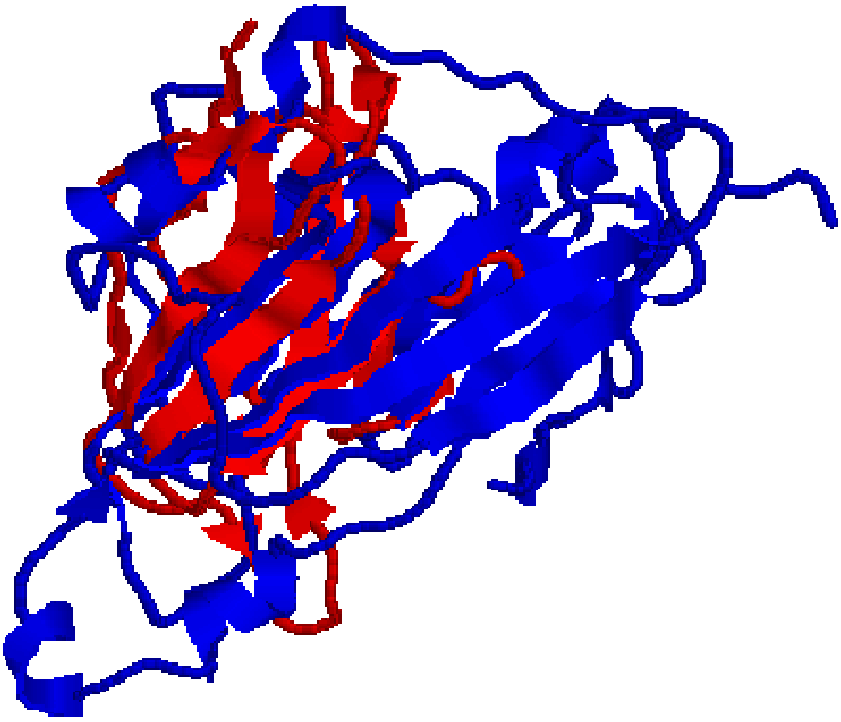

Figure 6: **Same SCOP family, different CATH architectures**: d1ub6l1 (b.1.1.1) / 1ub6L01 (2.60.40.10) vs. d1l2gc_ (b.1.1.1) / 1l2gC00 (2.70.230.10)

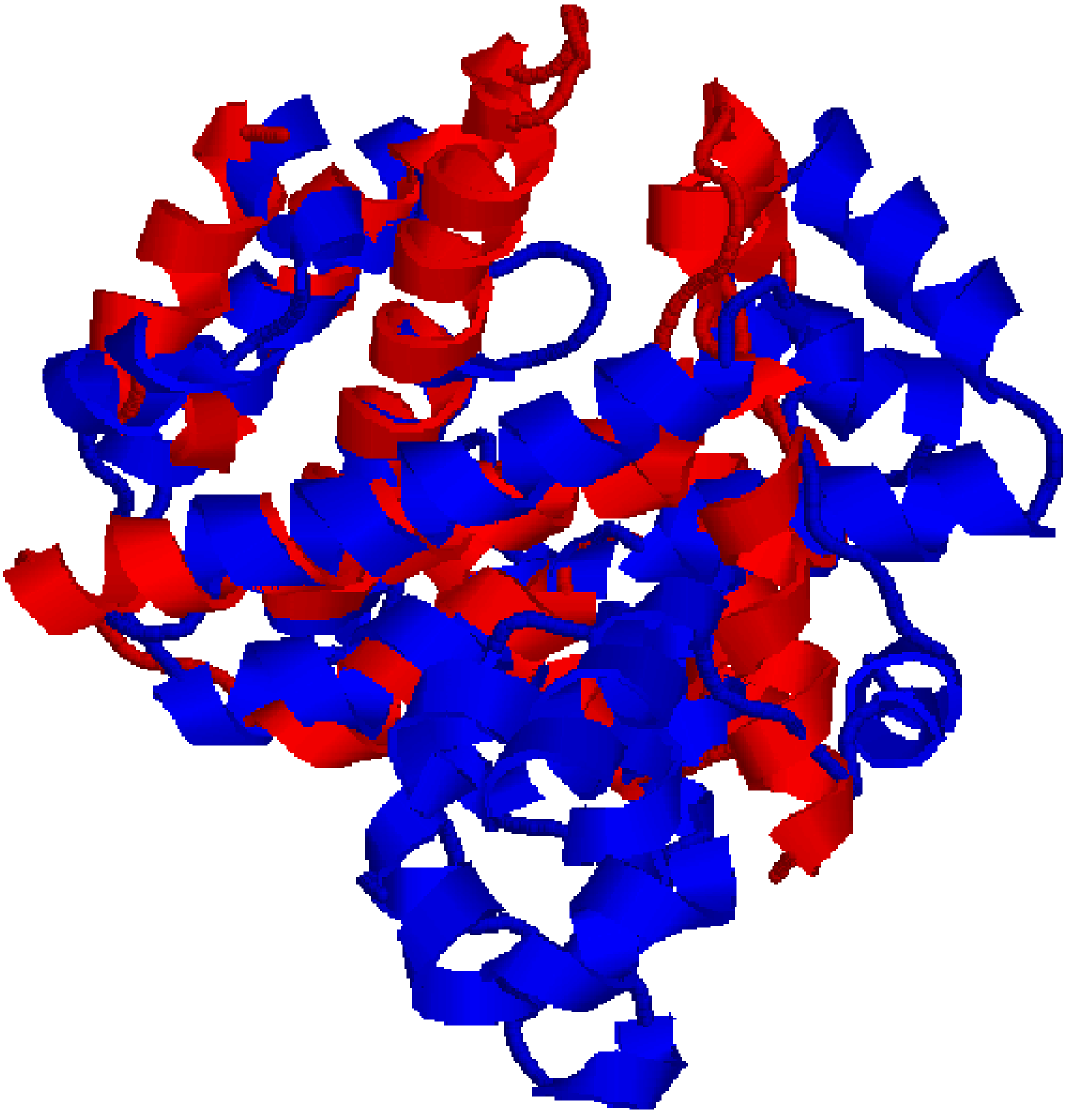

Figure 7: **Same SCOP superfamily, different CATH topologies**: d1xx7b_ (a.211.1.1) / 1xx7B00 (1.10.3210.10) vs. d1vqrd_ (a.211.1.3) / 1vqrD00 (1.10.3150.10)

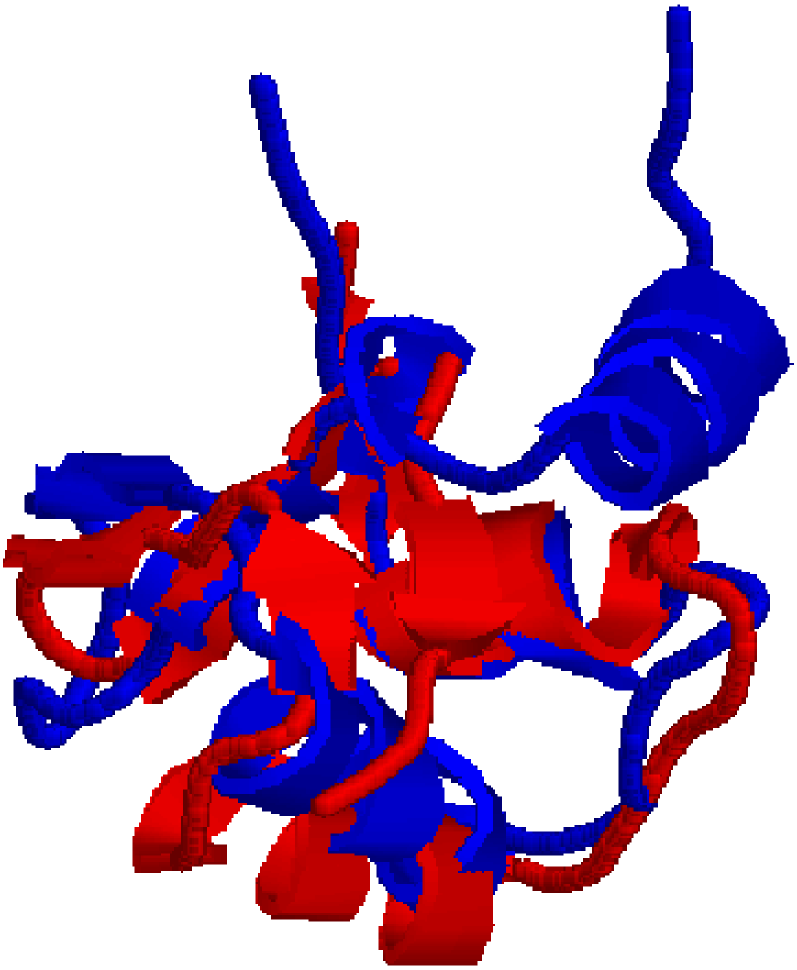

Figure 8: **Same SCOP family, different CATH topologies**: d1rh6b_ (a.6.1.7) / 1rh6B00 (1.10.1660.20) vs. d1g4da_ (a.6.1.7) / 1g4dA00 (1.10.10.10)

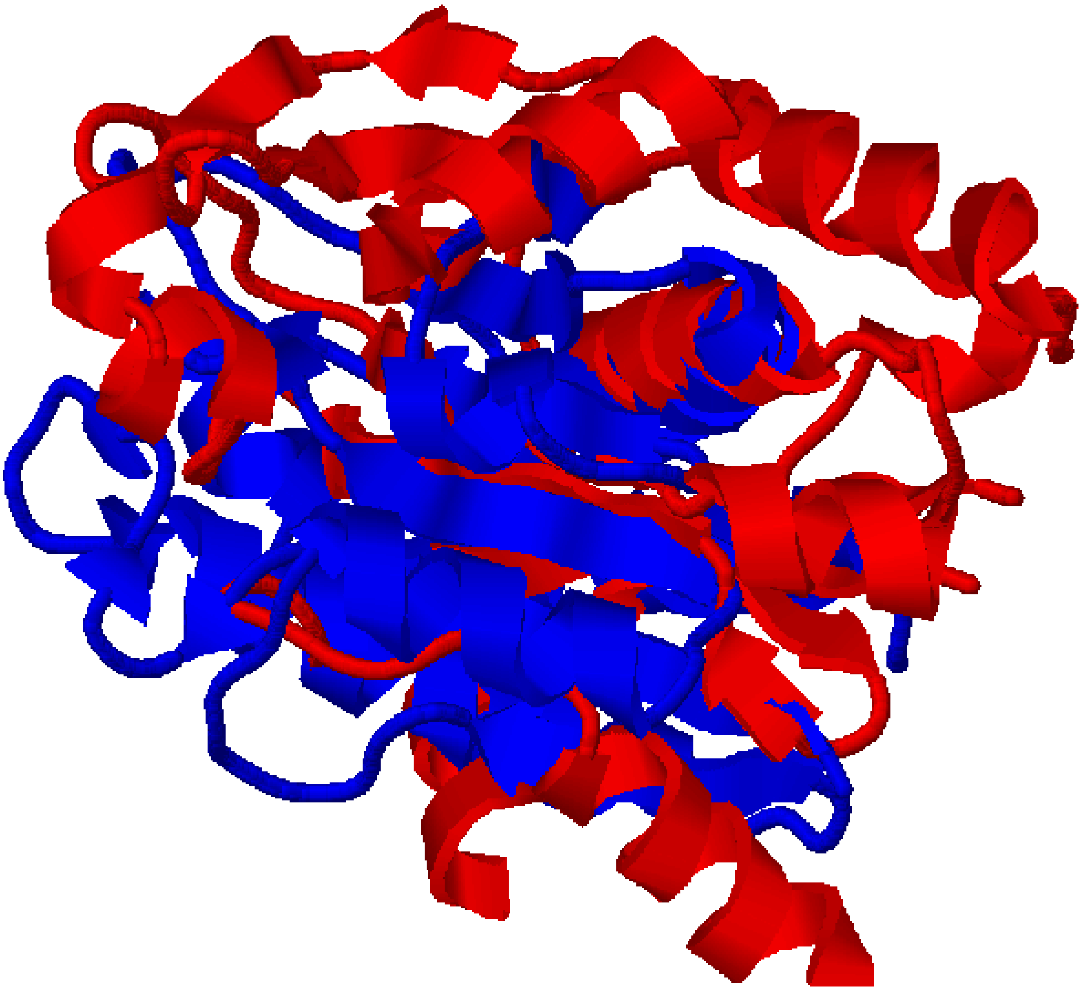

Figure 9: **Different SCOP classes, same CATH topology**: d1jwqa_ (c.56.5.6) / 1jwqA00 (3.40.630.40) vs. d1kzfa_ (d.108.1.3) / 1kzfA00 (3.40.630.30)

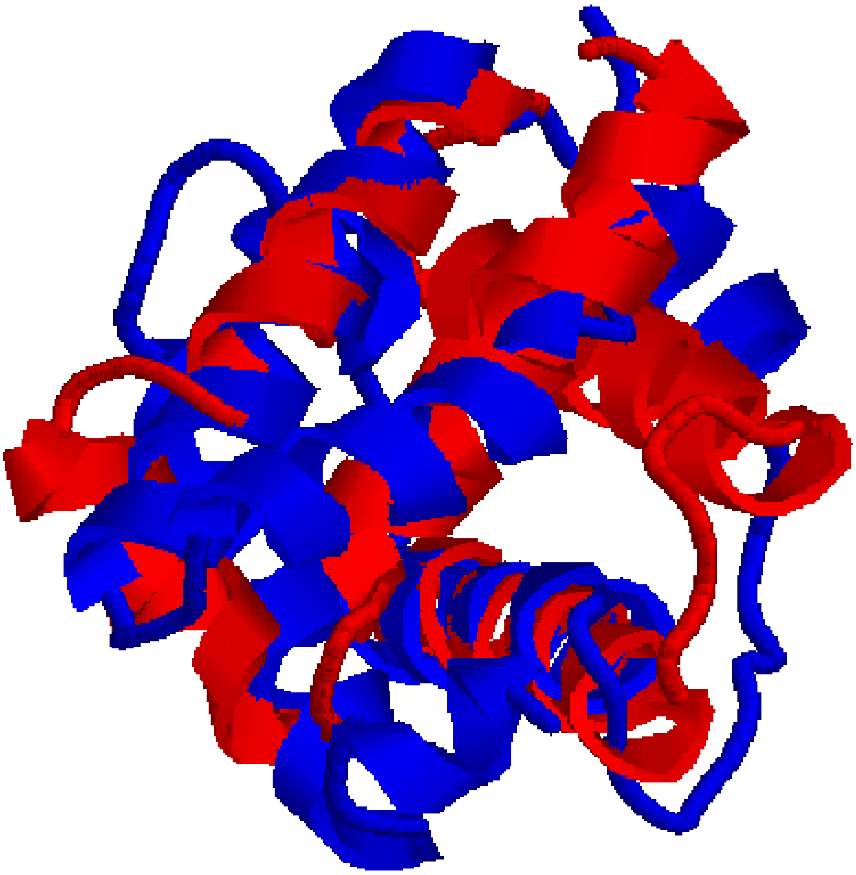

Figure 10: **Different SCOP folds, same CATH topology**: d1dlwa_ (a.1.1.1) / 1dlwA00 (1.10.490.10) vs. d1ny9a_ (a.181.1.1) / 1ny9A00 (1.10.490.50)

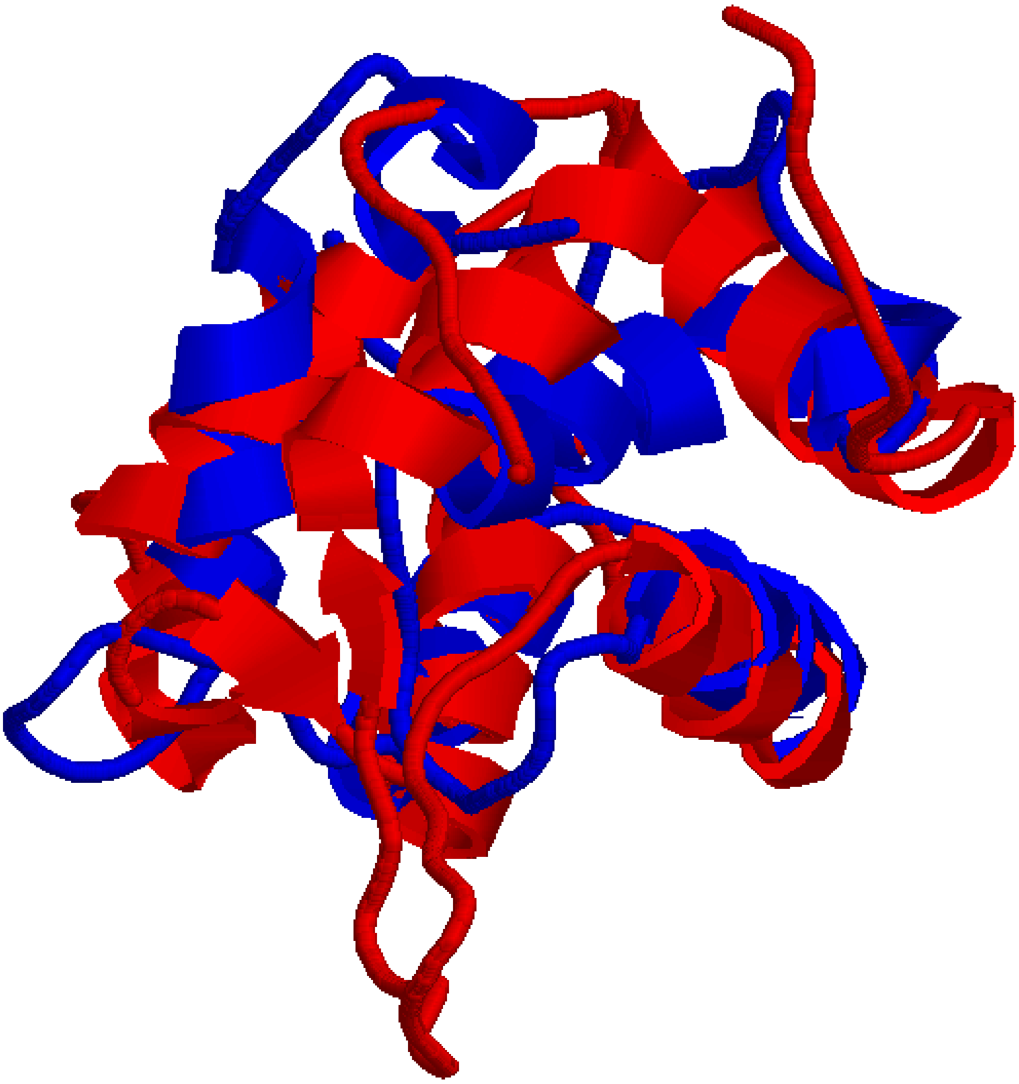

Figure 11: **Same SCOP superfamily, different CATH homologous superfamilies**: d1qjta_ (a.39.1.6) / 1qjtA00 (1.10.238.10) vs. d1tuza_ (a.39.1.7) / 1tuzA00 (1.10.238.110)

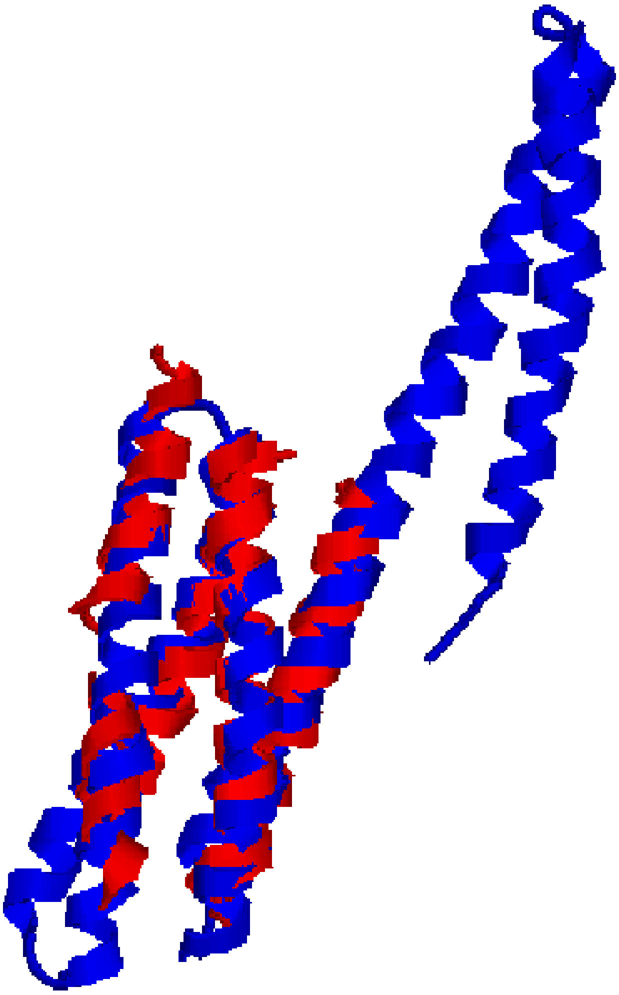

Figure 12: **Same SCOP family, different CATH homologous superfamilies**: d1l7cb2 (a.24.9.1) / 1l7cB02 (1.20.120.270) vs. d1dova_ (a.24.9.1) / 1dovA00 (1.20.120.230)

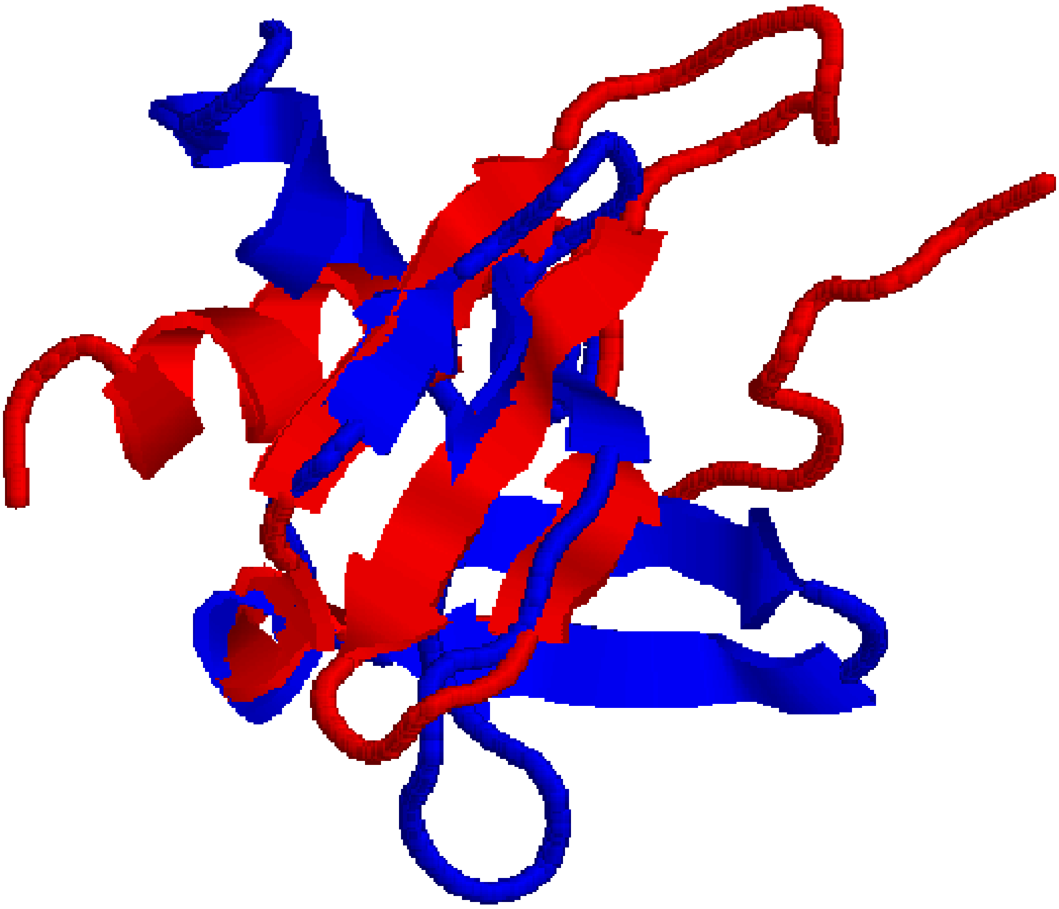

Figure 13: **Different SCOP classes, same CATH homologous superfamilies**: d1bbxd_ (b.34.13.1) / 1bbxD00 (2.40.50.40) vs. d1rhpa_ (d.9.1.1) / 1rhpA00 (2.40.50.40)

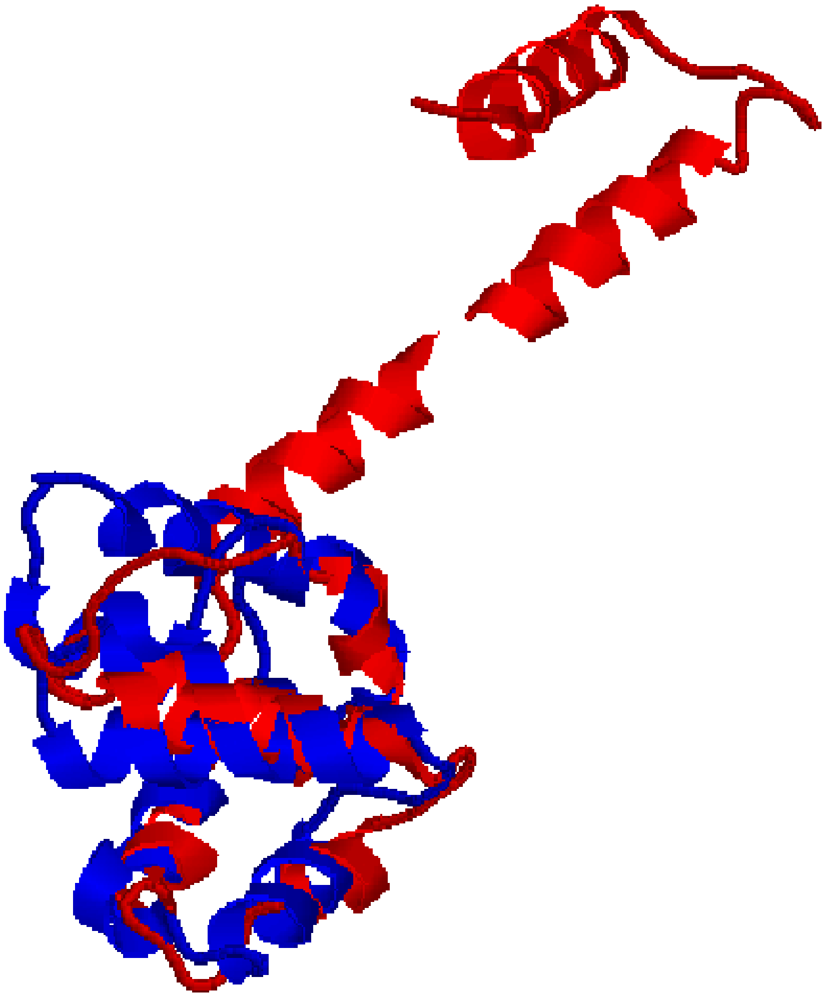

Figure 14: **Different SCOP folds, same CATH homologous superfamilies**: d1t3wb_ (a.236.1.1) / 1t3wB00 (1.10.860.10) vs. d1b79b_ (a.81.1.1) / 1b79B00 (1.10.860.10)

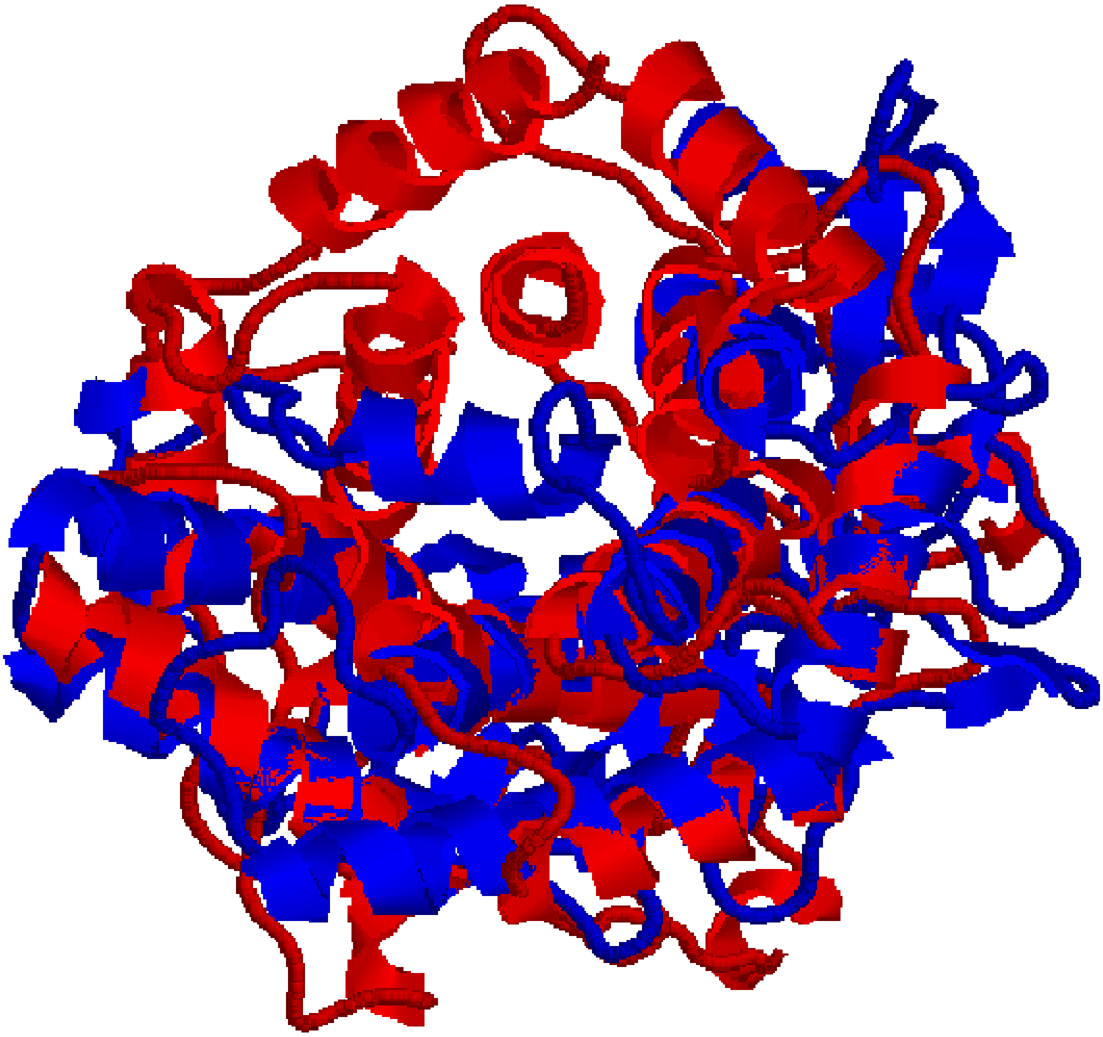

Figure 15: **Different SCOP superfamilies, same CATH homologous superfamilies**: d1o6rb1 (a.102.4.2) / 1o6rB01 (1.50.10.20) vs. d1gxna_ (a.102.5.1) / 1gxnA00 (1.50.10.20)
